# Monomeric C-Reactive Protein and Cerebral Hemorrhage: From Bench to Bedside

**DOI:** 10.3389/fimmu.2018.01921

**Published:** 2018-09-11

**Authors:** Mario Di Napoli, Mark Slevin, Aurel Popa-Wagner, Puneetpal Singh, Simona Lattanzi, Afshin A. Divani

**Affiliations:** ^1^Department of Neurology and Stroke Unit, San Camillo de' Lellis General Hospital, Rieti, Italy; ^2^Healthcare Science, Manchester Metropolitan University, Manchester, United Kingdom; ^3^Department of Neurology, University of Medicine Essen, Essen, Germany; ^4^Center of Clinical and Experimental Medicine, University of Medicine and Pharmacy, Craiova, Romania; ^5^Department of Human Genetics, Punjabi University, Patiala, India; ^6^Neurological Clinic, Department of Experimental and Clinical Medicine, Marche Polytechnic University, Ancona, Italy; ^7^Department of Neurology, University of Minnesota, Minneapolis, MN, United States; ^8^Department of Neurosurgery, University of Minnesota, Minneapolis, MN, United States

**Keywords:** CRP, inflammation, stroke, intracerebral hemorrhage, SAP, outcomes, risk assessment, biomarkers

## Abstract

C-reactive protein (CRP) is an important mediator and a hallmark of the acute-phase response to inflammation. High-sensitivity assays that accurately measure levels of CRP have been recommended for use in risk assessment in ischemic stroke patients. Elevation of CRP during the acute-phase response in intracerebral hemorrhage (ICH) is also associated with the outcomes such as death and vascular complications. However, no association has been found with the increased risk of ICH. The aim of this review is to synthesize the published literature on the associations of CRP with acute ICH both as a risk biomarker and predictor of short- and long-term outcomes as well as its role as a pathogenic determinant. We believe before any clinical utility, a critical appraisal of the strengths and deficiencies of the accumulated evidence is required both to evaluate the current state of knowledge and to improve the design of future clinical studies.

## Introduction

Inflammation is recognized to play a major role in the pathogenesis of cerebrovascular disease (1, 2). C-reactive protein (CRP), which is produced in the liver, is a hallmark of the acute inflammatory response, and represents an extensively studied systemic marker of inflammation (3). The significance of elevated CRP as a marker of inflammation in the clinical setting has been suggested in the literature (4–6). Notably, over recent decades CRP has been the focus of an intense investigation to explore its role in the setting of intracerebral hemorrhage (ICH) and currently is proposed as a risk assessment tool and prognostic marker (7).

The aim of this review is to synthesize the available literature examining the association of monomeric CRP with acute ICH as a risk assessment biomarker and predictor of short- and long-term outcomes as well as its role as a pathogenic determinant.

## Structure and function of CRP

CRP and serum amyloid P (SAP) component are the main pentraxins (PTX) in humans. PTX represent an evolutionarily conserved family of proteins mostly involved in immunological responses. They have a unique pentameric structure and bind to their ligands in a calcium-dependent manner (8, 9). CRP and SAP are soluble pattern-recognition proteins of pathogenic bacteria or damaged cells and, therefore, interact with the complement pathway and Fcγ receptors to activate the innate immune system (10, 11). CRP, also known as PTX1, primarily exists in two structurally and functionally independent forms: (1) net anti-inflammatory, serum-associated native pentameric CRP (npCRP), and (2) pro-inflammatory tissue-associated, monomeric CRP (mCRP) (10, 12).

## Definition of npCRP/mCRP

npCRP is an acute phase PTX produced mainly by the liver in response to inflammatory infection in humans. It is composed of five none-covalently linked identical subunits. It is plasma-soluble, and its concentrations are linked to level of systemic inflammatory response. Serum npCRP concentrations have been shown to increase from <3 to >100 μg/ml within 8–72 h from the onset of an inflammatory response (13, 14). Its physiological role is to bind to phosphocholine, expressed on the surface of dead or dying cells, stimulating the activation of the complement system via the C1Q complex.

mCRP is a monomer of the parent molecule npCRP. mCRP is mainly tissue-associated, as its formation requires a conformational change that renders the protein largely insoluble (15, 16). The circulating 115 kDa npCRP homopentamer irreversibly dissociates into 23 kDa monomers upon binding to pathogenic membranes, damaged or apoptotic cell membranes, activated platelets, or blood-derived microparticles (MPs). Due to contact with cells and tissue, it converts to the monomeric-biological form where it can remain chronically. Specifically, on contact with cell membranes, npCRP undergoes an intermediate conformational change through hydrophobic insertion, maintaining pentameric status but acquiring the properties of mCRP, this is followed by loss of the pentameric form by breakage of the intrasubunit disulphide bond. The reduced form of mCRP (rmCRP), where the intra-subunit disulphide bridge is broken (e.g., on contact of reducing agents such as thioredoxin), binds strongly to the cholesterol binding motif of membrane lipid rafts and actively stimulates cell signaling, following unlocking of the lipid raft interaction motif through multiple mechanisms (17). Amongst others, rmCRP/mCRP has been shown to stimulate inflammation directly, induce aberrant angiogenesis and promote platelet activation and aggregation. Importantly, standard clinical high sensitive (hs)-CRP assays cannot detect mCRP or urea-solubilized mCRP on MPs. hsCRP assays, therefore, can only measure the portion of total CRP in plasma that is in the soluble pentameric form (soluble npCRP). Currently, only by using flow cytometry and antibodies it is possible to measure both forms of CRP (npCRP and mCRP) on MPs to revealing a compartment of CRP not currently measured with hs-CRP technique (18).

## Role of CRP in inflammation

CRP shows unique properties amongst the PTX family. Thiele et al. (19) published information showing that dissociation of pentameric to monomeric CRP localizes and aggravates inflammation. This work provides *in vivo* evidence of a powerful pro-inflammatory effect of mCRP in striated muscle, atherosclerotic plaque and infarcted myocardium (human and rat tissues). Agrawal et al. (20, 21) reported that in an *in vitro* acidic solution, npCRP was able to bind to various types of proteins with altered conformations in a calcium-independent manner. Taking into account acidic pH at inflammatory sites, npCRP protects against toxic conditions caused by protein misfolding and aggregation maintaining extracellular proteostasis of the inflammatory site by inhibiting the aggregation of unfolded/misfolded proteins (22). In addition, a dramatic increase in expression of mCRP has been observed in blood vessels of damaged brain regions. Furthermore, mCRP is highly angiogenic, unlike the native molecule, and may affect tissue survival and development by influencing vascularization and remodeling (23). Krupinski et al. (24) demonstrated a strong expression of mCRP within microvessels with unstable plaques whilst normal looking arteries and stable fibrous lesions contained a significantly lower expression. The mCRP was mainly associated with inflammatory cells and newly formed angiogenic microvessels within unstable plaque regions (24). This suggested that mCRP may have a pathological role in the development of unstable atherosclerosis and/or increased risk of plaque thrombosis. mCRP specifically increases the activation of the inflammation both *in vitro* and *in vivo*, getting deposited chronically within the brain after ICH (25), and may play a role in perpetuating neuroinflammation after brain injury.

The liver is the primary site of CRP synthesis in humans. However, it is also expressed, albeit at low levels, in the CNS. In the brain, mCRP is produced in large quantities after an ischemic insult and in response to inflammation, becoming indefinitely attached to cellular components and within the extracellular matrix (ECM) (26). After ICH onset, CRP could be clearly localized inside blood vessels and in the cytoplasm of activated astrocytes and neurons within the perihematoma regions (27). CRP has also been detected in a number of neurodegenerative diseases like Alzheimer's disease (AD) (28–31), amyotrophic lateral sclerosis (32), and multiple sclerosis (33). Using unspecific and specific CRP antibodies against mCRP, immunoreactivity has been detected in the neurofibrillary tangles of AD patients (31) and elevated CRP concentrations have been associated with increased risk of developing dementia in older people. Increased concentrations of serum CRP have recently been associated with cerebral microstructural disintegration, suggesting an involvement of CRP in silent stroke-associated vascular dementia and, possibly, hemorrhagic stroke (27, 34). However, these findings do not exclude the systemic origin of CRP. It has been shown that CRP may reach the brain tissue from the circulation by activating the endothelial contractile machinery in both an *in vitro* blood-brain barrier (BBB) model and in an isolated whole brain preparation (35, 36). In summary, these data are consistent with the hypothesis that npCRP may be a member of extracellular chaperones protecting against toxic conditions caused by protein misfolding and aggregation in acidic inflammatory environments. npCRP dissociates to mCRP at sites of inflammation, which is then deposited in brain parenchima at the level of ECM and via its proinflammatory effects acts to amplify and localize inflammation.

## npCRP-mCRP cellular binding and mechanism of action

mCRP binds to macrophages via integrin α5β3 and subsequent AKT signaling inducing a pro-inflammatory status and chemotaxis (37). In addition, the immuno-modulatory effect of CRP is also mediated through its ability to interact with FcyRs (similar to the G class of IgGs), and to stimulate phagocytic cells (e.g., macrophages). CRP acts as an opsonin for *N. meningitides* and other bacteria. Therefore, as CRP-opsonized bacteria showed increased uptake by human macrophages and neutrophils, this could be useful regarding potential clearing mechanisms via enhanced macrophage engulfment and phagocytosis after antibody binding. Reduction of the intra-subunit disulfide bond which occurs on cellular contact (after calcium-dependent npCRP binding), allows unlocking of the lipid raft interaction motif enhancing significantly the cellular uptake and signal activation in macrophages/endothelium (38, 39). In endothelial cells, mCRP and specifically rmCRP is known to bind to membrane-associated enriched lipid rafts and stimulate cellular activation through phospholipase C, MAP kinase, and NF-κB (39). In addition, as previously shown in a monocyte cell line (37), integrins may also mediate the binding of monocytic cells and other cell types via α5β3 integrin binding to mCRP, and mediate pro-inflammatory signaling upon binding to mCRP.

## Anatomo-pathological study in ICH

**Figure 1:**
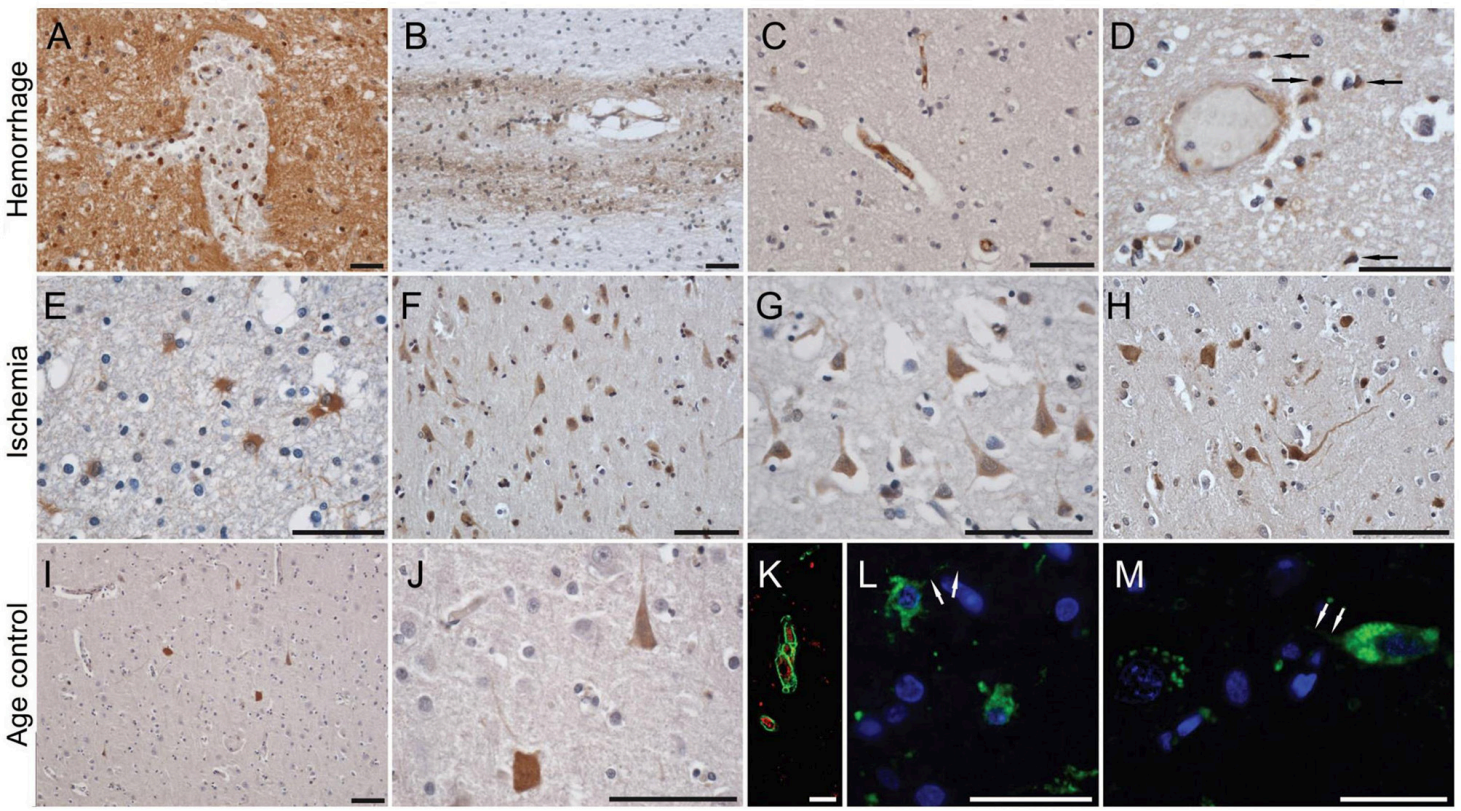
C-reactive protein staining in human brain after hemorrhagic and ischemic stroke and in normal aged control subjects. In the perihematoma areas, a diffuse neuropil staining was present, together with almost all cells' silhouettes taking up the stain **(A)**. Further away from the hemorrhagic core, the diffuse staining pattern diminished, but with some neurons clearly retaining an affinity for the antibody. Although distant from the hemorrhage, white matter fiber tracts showed relatively intense staining for CRP, especially those surrounding intracallosal vessels **(B)**. Focal intravascular staining sometimes was observed **(C)**, and only on occasion were microglia-like cells immunoreactive for CRP **(D)**. By immunofluorescence, CRP could be clearly localized inside blood vessels **(K)**, and in the cytoplasm of activated astrocytes **(L)** and neurons **(M)**. On the contrary, in the region immediately surrounding an ischemic area, a high number of gemistocytic astrocytes was noted **(E)**, while further away from the lesion core numerous neuronal silhouettes were observed **(F–H)**. On occasion, diffuse staining could be noted along the white matter tracts and blood vessels. In the control brain, and in the hemispheres contralateral to the lesion, only occasional neuronal silhouettes were stained, with their respective densities being clearly lower compared to the lesioned (ipsilateral) hemispheres **(I,J)**. Scale bars, 20 μm. Reproduced with permission from (27).

**Figure 2:**
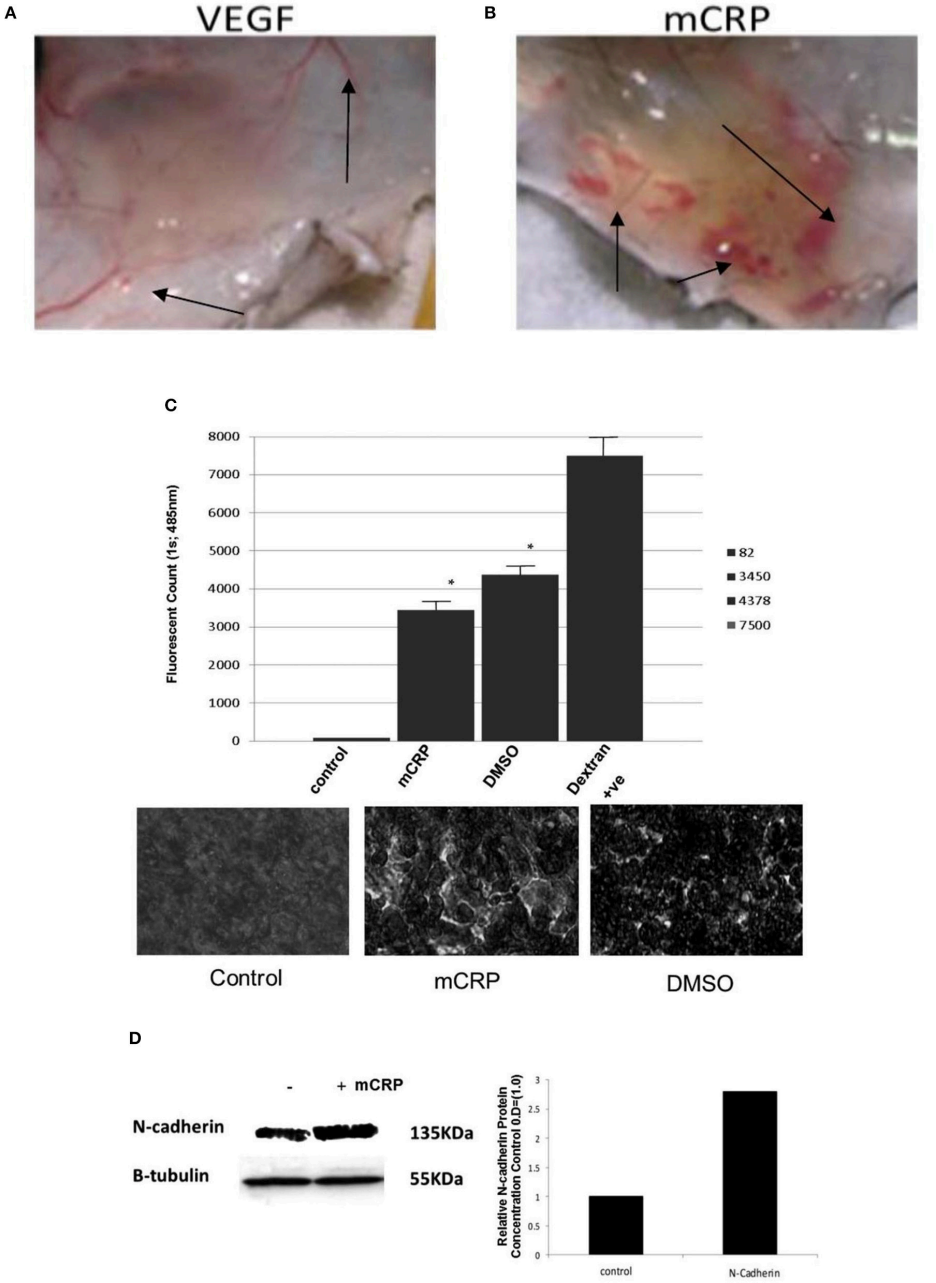
mCRP increases vascular permeability and aberrant angiogenesis. Top shows hemorrhagic blood vessels produced within a matrigel murine skin implant-compared with normal vessels after VEGF incorporation. Dorsal matrigel implants containing mCRP (10 μg/ml; 72 h) produced strong and visible hemorrhagic angiogenesis (**B**; arrows) compared with a typical, normal looking vascular response seen in the presence of VEGF (**A**; 25 ng/ml). In **(C)** the graph shows a significant increase in monolayer permeability in the presence of mCRP (10 μg/ml; 8 h) using a Millipore-based filter assay, similar to that produced by 10% DMSO (*p* < 0.01 increase in FITC dextran penetrating the monolayer in the presence of either mCRP or the positive control DMSO), and lighter regions in the images shown indicate areas of increased permeability. Lower images show intact control monolayer (left); mCRP-treated monolayer and (right) DMSO-treated monolayer showing enhanced permeability and surface damage. Lighter regions in the images shown indicate areas of increased permeability. **(D)** Expression of adhesion molecules was examined in BAEC treated with mCRP (10 μg/ml; 24 h). NCAM expression was increased by approximately 2.8-fold whilst VCAM, ICAM and integrins were not affected (data not shown). β-tubulin was used as the house keeping control (gel and bar chart shown). Reproduced with permission from (34).

Published data has shown that mCRP is strongly expressed in the brain parenchyma (neuronal nucleus and cytoplasm and angiogenic microvessels co-localized with CD105) of patients following ischemic stroke in the damaged core and penumbral regions (26). The mCRP remained visible and in significant quantities several months after the event. mCRP levels were also increased in brain tissue parenchyma after ICH (27), and was present in large amounts in perihematomal regions and within neurons and glia of patients who died within 12 h of spontaneous ICH (7, 27). Circulating levels of npCRP also predicted hematoma growth and outcome. mCRP was abundant in the walls of microvessels from the perihematoma region of the metabolic penumbra of patients who died soon (2 days) after stroke (Figure 1). mCRP may be responsible for increases in vascular permeability and aberrant angiogenesis leading to vessel structural instability and hemorrhagic conversion after stroke. *In vivo*/*ex-vivo* effects on vascular development include the findings that NCAM becomes over-activated, linked to pathological, aberrant angiogenesis, the FITC-dextran permeability assay showed that mCRP increased monolayer permeability, sprouting angiogenesis assays and gap junction spacing between cells, whilst dorsal matrigel implants containing mCRP produced hemorrhagic lesions (while nCRP and VEGF did not show a similar effect; see Figure 2).

## Epidemiological studies of CRP and ICH risk

Spontaneous ICH accounts for approximately 20% of all strokes, and it is characterized by high rates of mortality and residual disability among survivors (43, 44). Furthermore, unlike ischemic stroke, ICH typically has no identifiable premonitory signs or symptoms. Accordingly, the identification of individuals at risk of ICH, prior to the occurrence of the event, represents a striking clinical issue. On these grounds, older age, male sex, hypertension, diabetes mellitus, alcohol intake abuse and smoking have been associated with higher risk of ICH (45). Nonetheless, predictive models based upon conventional risk factors are still inadequate and, as such, there is a need for developing early preventive strategies. Low-grade inflammation is increasingly recognized as a key player in the pathophysiology underpinning many different medical conditions. Serum biomarkers related to increases in systemic inflammatory activity are significant predictors of cardiovascular diseases (CVD) and mortality (46). In this respect, the serum CRP concentration has close associations with the risk of coronary heart disease, ischemic stroke, and vascular mortality (47). On the contrary, there is no evidence providing a clear-cut relationship with the risk of ICH. In the last decade, a number of epidemiological studies across multiple ethnicities have been conducted, but none demonstrated a meaningful link between circulating CRP levels and ICH risk.

Based on 6,430 participants as part of the elderly population-based Rotterdam Study, Bos et al. (48). evaluated whether the risk of stroke varied with baseline CRP serum levels and whether that can helpful in the prediction of risk in individual stroke. During an average follow-up of 8.2 years, 498 first-ever strokes occurred, of which 51 (10%) were sub-classified as hemorrhagic. CRP levels were significantly associated with incident stroke and ischemic stroke, but not with ICH. The sex-specific hazard ratios for the associations between one standard deviation (SD) increase in logarithmically transformed CRP and ICH were 1.36 (0.87–2.14) and 0.81 (0.57–1.17) in men and women, respectively.

In a 12-years follow-up examination of a Japanese population in the town of Hisayama, from December 1988 through to November 2000, Wakugawa et al. (49) demonstrated that elevation of serum CRP levels was an independent risk factor for future ischemic stroke in men, whereas no association between serum CRP levels and the risk of future hemorrhagic stroke in either sex was observed. The age-adjusted incidence rates of first-ever hemorrhagic stroke according to quintiles of baseline serum CRP were 2.4, 1.1, 2.2, 1.9, and 2.7 per 1,000 person-years for men, and 1.1, 2.6, 1.0, 1.3, and 1.6 per 1,000 person-years for women with no significant trends in either sex.

Associations of CRP levels with risks of total stroke and its subtypes were examined in the Circulatory Risk in Communities Study (49), prospective nested case-control study of 13,521 Japanese men and women aged 40–85. A total of 261 incident strokes were identified, of which 67 were ICH and 29 were subarachnoid hemorrhage (SAH). After adjustment for known cardiovascular risk factors, CRP predicted the incidence of total and ischemic strokes, while no associations were associated with the risk of hemorrhagic stroke.

The role of hemostatic and inflammatory indices as predictors of ICH was addressed by analyzing data from the Cardiovascular Health Study (50), a randomly selected population-based cohort study from four US communities, which recruited 5,201 participants aged 65 years or older. Among the considered biomarkers, fibrinogen and von Willebrand factor but not CRP, factor VII, and white blood cell count were significantly associated with incidence of ICH.

In a prospective, population-based study nested within the Northern Sweden Cohorts, Andersson et al. (40) explored the role of CRP as a determinant of first-ever stroke and the relationships between the 1444C > T polymorphism, CRP levels, and stroke. CRP concentrations were categorized as <1, 1–3, and >3 mg/L for low, average, and high risk, respectively. Three hundreds and eight cases of ischemic stroke, 61 cases of ICH with time from ischemic and hemorrhagic strokes were 52 and 50 months, respectively. A total of 749 matched referents were defined as control. In a multivariate model including traditional risk factors, CRP was significantly associated with the risk of having a first ischemic but not ICH [OR for the highest CRP group: 2.58 (1.74–3.84) for ischemic stroke and 0.97 (0.30–3.11) for ICH]. Additionally, the CRP 1444 (CC/CT vs. TT) polymorphism was associated with plasma levels of CRP but not with ischemic stroke or ICH.

CRP levels and risk of mortality from CVD were investigated as part of the Japan Collaborative Cohort Study for evaluation of cancer risk (51). A total of 39,242 subjects of 40–79 years of age provided serum samples at baseline between 1988 and 1990; controls were matched for sex, age, residence area and year of serum storage. During the 13-years follow-up, the total strokes cases comprised of 214 hemorrhagic (119 ICH and 95 SAH) and 294 ischemic strokes. Higher serum CRP levels were associated with higher mortality from CVD, but not ICH. Multivariable odd ratios for one SD increment of CRP were 1.36 (0.75–2.48) in men and 1.50 (0.99–2.28) in women. The impact of CRP on risk of stroke, stroke subtypes, and ischemic heart disease in middle-aged Japanese was further explored in a prospective, population-based Study (52). A total of 1,341 CVD events occurred between 1990 and 2007 among subjects included in the study, including 494 hemorrhagic strokes (344 ICH and 150 SAH). The main finding was the lack of any association between CRP and risk of hemorrhagic stroke.

A longitudinal study from June 2006 to October 2007 based on the Kailuan community, in Tangshan city, included 90, 517 Chinese adults, with no history of stroke or myocardial infarction (MI) at baseline (53). During a total follow-up of 362,163 person-years and a median of 49 months per participants, 1,472 incident strokes were documented, of which 383 (26.0%) were ICH and 40 (2.7%) were SAH. The age- and sex-standardized incidence per 1,000 person-years of ICH increased with CRP concentrations, being 3.04, 4.36 and 4.61 (*p*-value for trend = 0.04) for <1, 1–3 and >3 mg/L groups, respectively. After adjusting for covariates, CRP concentrations ≥1 mg/L were associated with increased risks of all stroke but were not associated with ICH or SAH, regardless of hypertensive status.

A recent aggregate data meta-analysis considered prospective observational studies that enrolled participants from the general population, measured hs-CRP levels and reported adjusted estimates of stroke risk in at least three CRP categories (54). Four studies were included in the meta-analysis with a total of 40,002 subjects that 655 were ICH cases. Elevated CRP levels were associated with excessive risk of ischemic but not hemorrhagic stroke. The pooled adjusted risk ratio of hemorrhagic stroke comparing the highest with the lowest CRP category group was 0.82 (0.59–1.13; *p* = 0.217) in a fixed-effect model, with no evidence of heterogeneity. Although serum levels of CRP do not seem to predict the risk of future ICH, it is noteworthy that none of the aforementioned studies provided data on ICH etiology. The main causes of ICH (i.e., hypertension, lipohyalinosis of small vessels, and amyloid angiopathy) have different risk factor profiles and underlying pathophysiological pathways (55). Accordingly, the involvement of the inflammatory response and, in turn, the predictive role of serum CRP may differ according to the ICH subtype. Indeed, associations between serum CRP levels, cerebral small vessel disease, and lobar or deep micro-bleeds have been inconstantly reported highlighting the need for further investigations before drawing concrete conclusions (56–58). Finally, although hypertension and smoking may accelerate the development and growth of intracranial aneurysms (59), which are a main cause of SAH, the relationship between an atherosclerosis and the intracranial aneurysm is weak (60). This can explain the lack of a clear association between CRP levels and SAH. Finally, the lack of a significant association between ICH and CRP can also be due to clinical hs-CRP assays that only measure the portion of total CRP in plasma in the soluble pentameric form (18). The insoluble component of CRP, npCRP and mCRP, on MP is not currently measured in clinical settings that may have a relevant pathogenetic role that is different from soluble pentameric form in ischemic vascular disease (18).

## Cerebral hemorrhage and genetics of CRP

Several studies of CRP genetics have focused on genetic polymorphisms and development of CVD. The impact of genetic variants of CRP single-nucleotide polymorphisms (SNPs) such as rs1800947, rs1417938, rs1130864, and rs3093077 on circulating protein level and outcome has been recently assessed in a cohort of in-patients with CVD. It was found that both CRP level ≥5 mg/L and SNP rs1800947 of the CRP gene were independent risk factors for further adverse vascular events among patients with CVD within a 3 years follow-up (61).

The 717A > G polymorphism, which is located in the promoter region of the CRP gene, has been associated with ischemic stroke and it was an independent predictor of cerebral ischemia among Chinese population. Although in hemorrhagic stroke patients the frequency of haplotype H5 (A-T-C) was lower compared to controls, it was not an independent protective factor against cerebral hemorrhage. Accordingly, H3 haplotype (G-C-C) could be an independent risk marker for ischemic stroke, and the H5 haplotype (A-T-C) could act as a prognostic marker of hemorrhagic stroke (38, 62).

CRP levels differ in individuals and this inter-individual variation is genetically controlled with a substantial heritability score (63, 64). The CRP gene that controls its blood levels is localized at chromosome position 1q21–1q23, spans 1.9 kb and exhibits two exons. Twenty-nine different polymorphisms have been assessed within the CRP gene, however, so far there is a paucity of robust studies clarifying the genetic consequences of CRP gene vis-à-vis ICH. Four genetic studies have been conducted hitherto, one of which has shown that haplotype ATC of CRP SNPs (rs2794521, rs3091244, and rs1205) is an independent prognostic marker for hemorrhagic stroke (38).

Another prospective population-based study on a Swedish cohort has examined the contribution of 1444C/T polymorphism of the CRP gene for the risk of ischemic stroke and ICH. Using a multivariate model including traditional risk factors, CRP levels were found to be significantly associated with ischemic stroke (OR 2.06, 95%CI 1.29–3.29), whilst the 1444C/T polymorphism failed to correlate with ICH (40). Das et al. (41) investigated the 1059G/C polymorphism of the CRP gene in relation to its contribution for the risk of ischemic and hemorrhagic stroke in an Indian population. Results of this study showed that CRP levels were increased significantly in hemorrhagic stroke patients (*p* < 0.001) when compared with controls, but the CRP gene polymorphism was not found to be associated with hemorrhagic stroke. A case-control study involving 236 hemorrhagic stroke patients in a community of Han Chinese population revealed that SNP rs3091244 (−286C/T/A) of the CRP gene was significantly associated with elevated CRP levels in male patients (42). Summarizing the above data (see Table 1), the studies investigating CRP gene polymorphism as the genetic determinant of ICH are inadequate. The inferences drawn from these studies can only be considered as suggestive rather than conclusive due to the unchecked false positive cases and lack of proper statistical power. More genetic studies in different populations after adjusting risk concomitants will enhance our knowledge regarding the contribution of the CRP gene and its underlying contribution to the prognosis and prediction of ICH.

**Table 1:** Genetic studies of CRP polymorphisms in intra-cerebral hemorrhage stroke patients.

**Population**	**CRP-SNPs**	**Sample size**	**Diagnostic criteria**	**Results**	**References**
Swedish	1444C/T (rs1130864)	Ischemic stroke: 308 ICH: 61	TOAST criteria	CRP levels were associated with ischemic stroke in multivariable model; however, 1444C/T polymorphism was not associated with any type of stroke.	(40)
Chinese	−757A/G (rs3093059), −717A/G (rs2794521), −286C/T/A (rs3091244), 2147C/T (rs1205)	Ischemic stroke:431 ICH: 67	CT and/or MRI	−717A allele showed protective effect for ischemic stroke. Haplotype H3 (GCC) influenced the risk for IS (OR 1.05; 95%CI 1.00–1.10, *P* = 0.04), whereas, haplotype H5 (ATC) influenced the risk for ICH (*P* = 0.0001).	(38)
Indian	1059G/C (rs1800947)	Ischemic stroke:200 ICH:200	CT and/or MRI	hsCRP levels were significantly increased (*P* < 0.001) in ICH patients, however, CRP gene polymorphism (rs1800947) did not correlate with CRP levels.	(41)
Chinese	−757A/G (rs3093059), −717A/G (rs2794521), −286C/T/A (rs3091244), 3′UTR-T/C (rs876537)	ICH: 236 Controls:993	CT and/or MRI	−286C/T/A (rs3091244) was significantly associated with higher hs-CRP levels whereas, rs2794521 showed negative correlation with its levels in ICH patients.	(42)

*IS, ischemic stroke; ICH, intracerebral hemorrhage*.

## CRP stratification in acute ICH: short and long-term prognosis

Early prediction of ICH outcome is a clinical priority to stratify patient risks, objectively design individual management, assess patient prognosis with reasonable accuracy, and standardize communication among healthcare providers. The evidence that ICH prognosis cannot be fully explained by traditional baseline hematoma features alone has prompted to look for adjunctive biochemical (65, 66), radiological (67, 68), clinical (67–70), and therapeutic (71, 72) variables that could characterize and inform the secondary-induced brain injury and improve outcome prediction.

The local and systemic inflammatory response that takes place shortly after the ICH onset can enhance the brain injury and post-stroke complications (73). Immune biomarkers, including fever, white blood cell count, neutrophil-to-lymphocyte ratio, interleukin-6 and fibrinogen have been shown to improve outcome prognostication (74–77). On these grounds, the specific relationship between CRP and short- and long-term ICH outcome has been also extensively explored over the past few years.

In a cohort of 399 patients presenting with primary or vitamin K antagonist (VKA)-associated ICH plasma CRP levels, measured within 6 h from onset were significantly associated with the occurrence of hematoma growth and neurological worsening (7). In the final model accounting for age, sex, VKA use, GCS score, ICH volume, midline shift, intra-ventricular hemorrhage, time of sampling and white blood cells count, CRP > 10 mg/L independently predicted hematoma growth [OR 4.71 (2.75–8.06); *p* < 0.0001] and neurological worsening [OR 2.70 (1.50–4.84); *p* = 0.0009), both of which increased the risk of mortality. Alexandrova et al. (78) examined 46 patients with ICH within 48 h after onset of symptoms and found admission serum CRP level to be a strong predictor of short-term fatal outcome. A 5.2% increase in the odds of first-week mortality was associated with an increase of CRP concentration by 1 mg/L. A prospective, multicenter, international, longitudinal study analyzed CRP kinetics and its association with short-term prognosis after ICH (27). A total of 223 patients were recruited, and CRP was evaluated at admission, 24, 48, and 72 h after symptom onset. Plasma CRP increased markedly from 48 to 72 h from admission, and the magnitude of the response was related to hematoma volume at later time points. Higher levels of CRP were independently associated with higher mortality and poor functional outcome at 30 days. From a clinical perspective, it is noteworthy to mention that serial CRP measurements during the acute ICH stages could provide different predictive utility: admission CRP values were only weakly related to mortality and did not predict functional status, CRP at 24 h was a better predictor of mortality and unfavorable outcome, and predictability improved further with the obtained CRP levels at 48 or 72 h that was stronger for mortality than for functional recovery (27, 79, 80).

CRP has also been demonstrated within neurons and glial cells around the hemorrhagic lesion in an immunostaining analysis of brain tissue of patients who died within 12 h of ICH (27). The early presence of CRP—which could reflect either the local synthesis triggered by the hematoma or the conversion of the circulating soluble pentameric form to its insoluble and monomeric form—further supports the active role of CRP in defining the extent of damage. The addition of CRP concentration to the ICH-score significantly increased the ability to predict 30-days mortality by about 8% (80). The net benefit in prognostic accuracy was greater in patients classified as being at low to moderate risk using the ICH-score alone compared with the highest risk patients, in whom the severity of ICH could reasonably be the major determinant of death, and the lowest risk category, in which comorbidities could represent the major determinants of prognosis. Acute phase reaction biochemical markers including CRP were significantly associated with a 3-months good prognosis [modified Rankin scale (mRS) score ≤2] in the bivariate analysis performed by Castellanos et al. (81) to identify outcome predicting variables in patients presenting with primary medium to large spontaneous hemispheric ICH within the first 12 h of symptom onset.

Löppönen et al. (82) identified 807 subjects who suffered primary ICH between 1993 and 2008 among the population of Northern Ostrobothnia, Finland, and extracted the CRP values within 24 h after symptom onset from the records. The analysis confirmed high serum CRP levels as an independent predictor of unfavorable 3-months outcome. The interconnections between infections, CRP levels, and ICH outcome were addressed by Diedler and colleagues (83). In a cohort of 103 patients with supratentorial hematoma, infections occurred during a hospital stay in 52 cases. Patients classified as having poor status (mRS > 2) at discharge had a higher incidence of infections and higher baseline (median 6.2 vs. <0.2 mg/dl; *p* = 0.001) and maximal CRP levels (median 79.6 vs. 7.6 mg/dl; *p* < 0.001) compared to patients with good prognosis. A similar pattern was also observed for the 1-year assessment [median baseline CRP 7.0 vs. 1.0 mg/dL (*p* = 0.006) and median maximal CRP 91.3 vs. 9.5 mg/dL (*p* < 0.001) for poor vs. good outcome]. The multivariate logistic regression model showed that maximal CRP levels were independent predictors of scanty functional outcome and mortality at discharge and long-term follow-up.

In a prospective study conducted at Sarawak General Hospital, Malaysia, from April 2013 to April 2015, 60 patients with a diagnosis of supratentorial ICH within 24 h after symptom onset were recruited to determine mortality and morbidity at 6 months (84). Admission and 72-h CRP levels were found to be the main determinants of 6-months functional outcome and overall survival, alongside baseline GCS, hematoma volume, and total leukocytes (84). The relationship between CRP and long-term ICH outcome was further explored in a prospective, multi-center, case-cohort study for the assessment of stroke risk factors, which enrolled 291 patients with first-ever stroke (196 ischemic and 95 hemorrhagic) between November 2000 and July 2001 from five medical centers in Hubei province in China (85). Patients were followed for 5 years, and the primary outcome included the occurrence of acute MI, ischemic or hemorrhagic stroke, and death. The mean plasma CRP levels in patients with and without endpoint events were 4.4 and 2.7 mg/L (*p* < 0.01), respectively, and the relative risk of death or vascular events during the 5-years follow-up was 3.01 (1.60–5.64; *p* = 0.001) in patients with CRP > 3 mg/L compared to patients with CRP < 1 mg/L.

## Conclusions

CRP seems to be an independent predictor of ICH outcome that is a reliable, easily accessible prognostic tool. The values of CRP concentrations for the prediction of ICH prognosis have been recognized in a variety of ethnic groups with conflicting results, so there is a possibility that it predicts different ICH phenotypes in different populations. The concentrations of CRP and its predication values on variety of stroke phenotypes are influenced by the ethnic genome background in the different racial groups, sex, and the environment. Based on the current report, CRP should be used while taking in mind these limitations. Furthermore, it is important to understand whether CRP polymorphisms affect the expression level or function of the CRP and whether single plays a key role or multiple polymorphisms act together. Finally, before routine use of CRP measurements in ICH prognostication, the clinical bioassays of circulating CRP should be refined. Actual hs-CRP assays do not reveal all the components of CRP subtypes. As discussed here, this aspect appears more relevant in ICH than in ischemic stroke. However, the inclusion of CRP subtype assays to aid in diagnosis or treatment of ICH relies on further studies. CRP does not simply reflect the strength of the acute-phase reaction but also correlates with the inflammatory response in the brain tissue. CRP can directly be involved with neuroinflammation and secondary-induced damage by activating the complement cascade and microglia, promoting leukocyte chemotaxis and adhesion molecule expression, stimulating cytokines release, inducing caspase-dependent apoptosis, BBB disruption, and brain edema formation. The downstream effects of mCRP within the damaged brain tissue can be blocked by preventing its cellular binding via lipid rafts and associated cell signaling soon after ICH onset. In this way, we can nullify the neurodegenerative cascade via a dual action of protecting neurons and preventing further re-bleeding thereby reducing the risk of hematoma expansion. This should be achievable through production of a specific blocking antibody, which could be delivered intra-arterially to bind to mCRP released into the extracellular matrix to phagocytic clearance of apoptotic cells from the brain parenchyma. The further understanding of the underlying signaling pathways may be useful to identify new targets for neuroprotection and develop future strategies for treatment of ICH.

## Author contributions

MD, MS, AP-W, PS, SL, and AD have designed the study, managed the work, and drafted the script.

### Conflict of interest statement

The authors declare that the research was conducted in the absence of any commercial or financial relationships that could be construed as a potential conflict of interest.
